# Response to Ruby et al: On a ‘failed’ attempt to manipulate conscious perception with transcranial magnetic stimulation to prefrontal cortex

**DOI:** 10.1016/j.concog.2018.07.011

**Published:** 2018-10

**Authors:** Daniel Bor, Adam B. Barrett, David J. Schwartzman, Anil K. Seth

**Affiliations:** a Department of Psychology, University of Cambridge, Cambridge CB2 3EB, UK; b Sackler Centre for Consciousness Science, University of Sussex, Brighton BN1 9QJ, UK; c School of Engineering and Informatics, University of Sussex, Brighton BN1 9QJ, UK

**Keywords:** Metacognition, Transcranial magnetic stimulation, Consciousness, Perception, Dorsolateral prefrontal cortex, Null result, Failed replication, Signal detection theory

## Abstract

Does disruption of prefrontal cortical activity using transcranial magnetic stimulation (TMS) impair visual metacognition? An initial study supporting this idea (Rounis, Maniscalco, Rothwell, Passingham, & Lau, 2010) motivated an attempted replication and extension (Bor, Schwartzman, Barrett, & Seth, 2017). Bor et al. failed to replicate the initial study, concluding that there was not good evidence that TMS to dorsolateral prefrontal cortex impairs visual metacognition. This failed replication has recently been critiqued by some of the authors of the initial study (Ruby, Maniscalco, & Peters, 2018). Here we argue that these criticisms are misplaced. In our response, we encounter some more general issues concerning good practice in replication of cognitive neuroscience studies, and in setting criteria for excluding data when employing statistical analyses like signal detection theory. We look forward to further studies investigating the role of prefrontal cortex in metacognition, with increasingly refined methodologies, motivated by the discussions in this series of papers.

## 1 Introduction

The role of prefrontal cortex (PFC) in visual awareness remains hotly debated (Boly et al., 2017, Bor and Seth, 2012, Odegaard et al., 2017, Tsuchiya et al., 2015). One way of addressing this topic is to disrupt prefrontal activity using (theta-burst) TMS, while examining any effects on subjective aspects of visual perception. An influential 2010 study took this approach, combining theta-burst TMS (tbTMS) with an innovative signal-detection-theory (SDT) analysis in a simple perceptual decision task (Rounis, Maniscalco, Rothwell, Passingham, & Lau, 2010). A major contribution of this study was to introduce the SDT quantity - *meta-d’* - which quantifies, in a principled and statistically unbiased way (assuming the model is true), how subjective reports (such as visibility or confidence ratings) discriminate the accuracy of first-order perceptual decisions (such as whether a stimulus is a square or a diamond). Rounis et al. found that tbTMS to dorsolateral PFC (DLPFC) selectively reduced visual metacognition, while first-order perceptual accuracy remained unchanged by design. This finding, if it stands up, provides strong evidence supporting a causal role for DLPFC in visual metacognition, which in turn support [at least on some theories, (Lau & Rosenthal, 2011)], a causal role in conscious perception.

However, attempted replications by our laboratory failed to find any reliable effect of tbTMS to DLPFC on visual metacognition (Bor, Schwartzman, Barrett, & Seth, 2017), concluding that, across studies, there was no reliable evidence for such an effect. This study in turn has been critiqued by authors from the original paper (Ruby, Maniscalco, & Peters, 2018), who go so far as to claim, on the basis of computational modelling, that the Bor et al. study in fact supports the findings in the original Rounis et al. study. We disagree with this surprising claim. We maintain that the results of our study should be understood following our interpretation in Bor et al.: that there is not (yet) good evidence that tbTMS to DLPFC disrupts visual metacognition. In substantiating our arguments, we encounter more general issues concerning what constitutes good practice in replication of cognitive neuroscience studies, and in setting criteria for excluding data when employing statistical analyses like signal detection theory (SDT). Discussion of these issues is, we believe, worthwhile beyond the current set of studies. We therefore welcome the continuation of this productive debate. Finally, we recognise that whether or not PFC is causally implicated in perceptual metacognition and conscious perception remains an important open question, and that other sources of evidence including lesion and neuroimaging studies will play an important role in addressing this question (Boly et al., 2017, Lau and Rosenthal, 2011, Odegaard et al., 2017).

## 2 Ruby et al.’s argument

In this spirit of open and constructive debate, the remainder of this paper describes our opinion on Ruby et al. (2018). Let us first summarise our understanding of their argument, the heart of which is statistical and computational. They focus on the fact that, in Bor et al. (2017), we excluded a substantial number of subjects from our analyses, in comparison to the original analyses in Rounis et al. (2010). In their view, our exclusions were not necessary since, according to computational simulations they performed, they did not affect false positive (FP) rates. Given that in Bor et al. we found a result which they claim was in line with Rounis et al., when we did *not* exclude subjects, they argue that we unnecessarily lowered our chances of finding a true effect (of TMS on metacognition) when we did apply our rigorous exclusion criteria. Ruby et al. then present a Bayesian analysis purporting to show that the data from Bor et al., support, rather than conflict with, Rounis et al.

We disagree with this claim, as we will explain.

Ruby et al. also selectively criticize some of the methodological changes we made, raising important issues about what constitutes an adequate replication. We take the opportunity here to revisit the reasons for these design decisions in the context of this broader concern. Finally, Ruby et al. appeal to evidence from a diverse range of alternative studies when discussing the overall question of the role of PFC in metacognition and consciousness. We do not think this appeal is relevant to the specific discussion about methods and analyses. Instead, it leads to further confusion since we do not claim our analyses undermine any of these alternative studies.

### 2.1 Subject exclusion, FP rates, and the value of ‘clean’ data

Ruby et al. motivate their simulations by saying that our main reason for subject exclusion was to avoid a high FP rate. Ruby et al. claim that excluding the subjects as we did in Bor et al. (2017) was ‘unnecessary’, on the basis of their computer simulations showing that FP rates are unaffected by such exclusions. Although we still maintain that including unreliable meta d’ results (“unreliable” meaning no longer measuring what it purports to, no longer a smooth, linear function, so that now a small change in underlying psychological components, such as hit rate, can lead to a large jump in the measure) may well increase FP rates (see below for reasons), this is a secondary issue, and is a misrepresentation of our reasoning, which is the critical point of disagreement between us.

We in fact performed these exclusions based on well-established theoretical arguments, elaborated in Barrett, Dienes, and Seth (2013) and fully described in Bor et al. Specifically, we excluded subjects when they had false alarm (FAR) or hit rates (HR) either less than 0.05 or greater than 0.95, at either the Type I (objective decision) or Type II (metacognitive) levels,^1^ in other words values that were numerically “extreme” by being close to or at the 0 or 1 limits. In Barrett et al. (2013), we showed that it is principled to apply these criteria because, otherwise, as we stated in the Bor et al. (2017) paper, “including such extreme results in the analysis is very likely to introduce instabilities [here referred to as “unreliabilities”] in measures reliant on type I and II SDT quantities, including type II d’ and especially various implementations of meta-d’ … Specifically, since the z function (i.e. the inverse of the standard normal cumulative distribution) approaches plus or minus infinity as HR or FAR tends to 0 or 1, SDT measures such as meta-d’ can take on extreme and highly inaccurate values with such inputs. In practice, we demonstrated from our data that unstable [in this commentary referred to as “unreliable”] meta-d’ – d’ values are significantly different from stable [reliable] values” (p. 15).

**Fig. 1:**
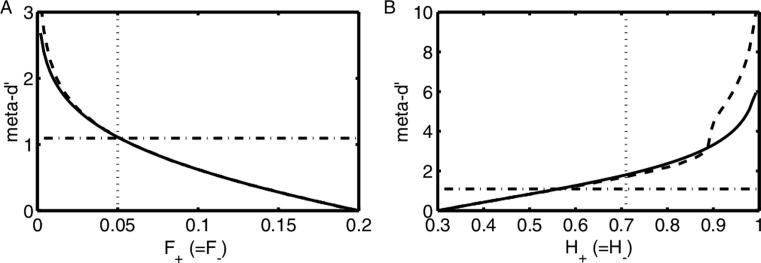
Simulated data demonstrating the behaviour of meta-d’ under systematic variation of Type II false alarm and hit rates. For both graphs, meta d’ balance (an analytical version of meta d’) is a solid line, and meta d’ SSE is the dashed line. Dashed-dotted lines show d’. The vertical dashed line in this simulation is the boundary where the measure becomes unreliable (clearly non-linear). (A) Shows meta-d’ against type II false alarm rate. (B) Shows meta-d’ against type II hit rate. Note that, particularly for meta d’ SSE, for high type II hit rates, meta d’ becomes unreliable. For parameter and model details, see Fig. 5 from Barrett et al. (2013). Reproduced with permission.

As this is such a critical point, let us rephrase it here: When hit rates or false alarm rates (HR/FARs) are *not* near 0 or 1, there is a roughly linear relationship between changes in HRs or FARs and meta d’. Such smooth, approximately linear relationships are good indicators of a reliable measure (i.e. one that accurately reflects the psychological process it is capturing, and where a small change in some psychological component, such as HR, will lead to a small change in the measure). However, when HRs or FARs become too big or too small (i.e. close to 0 or 1), a small change to them causes a big change in meta-d’. In other words, at these (numerically) extreme values, the relationship between changes in HRs or FARs changes from smooth and linear to non-smooth and non-linear, and the meta d’ measure is no longer reliable. This is clearly illustrated in Barrett et al. (2013) Fig. 5, reproduced here (see Fig. 1), where type II FARs approaching 0 or HRs approaching 1 cause meta d’ values to significantly deviate from their previously linear path. Note that the unreliability of the measure seems to be a particular issue for the SSE method, which was the main version of meta d’ used both in the Rounis et al. (2010) and our Bor et al. (2017) studies (in order to keep close to the Rounis et al. methods).

**Fig. 2:**
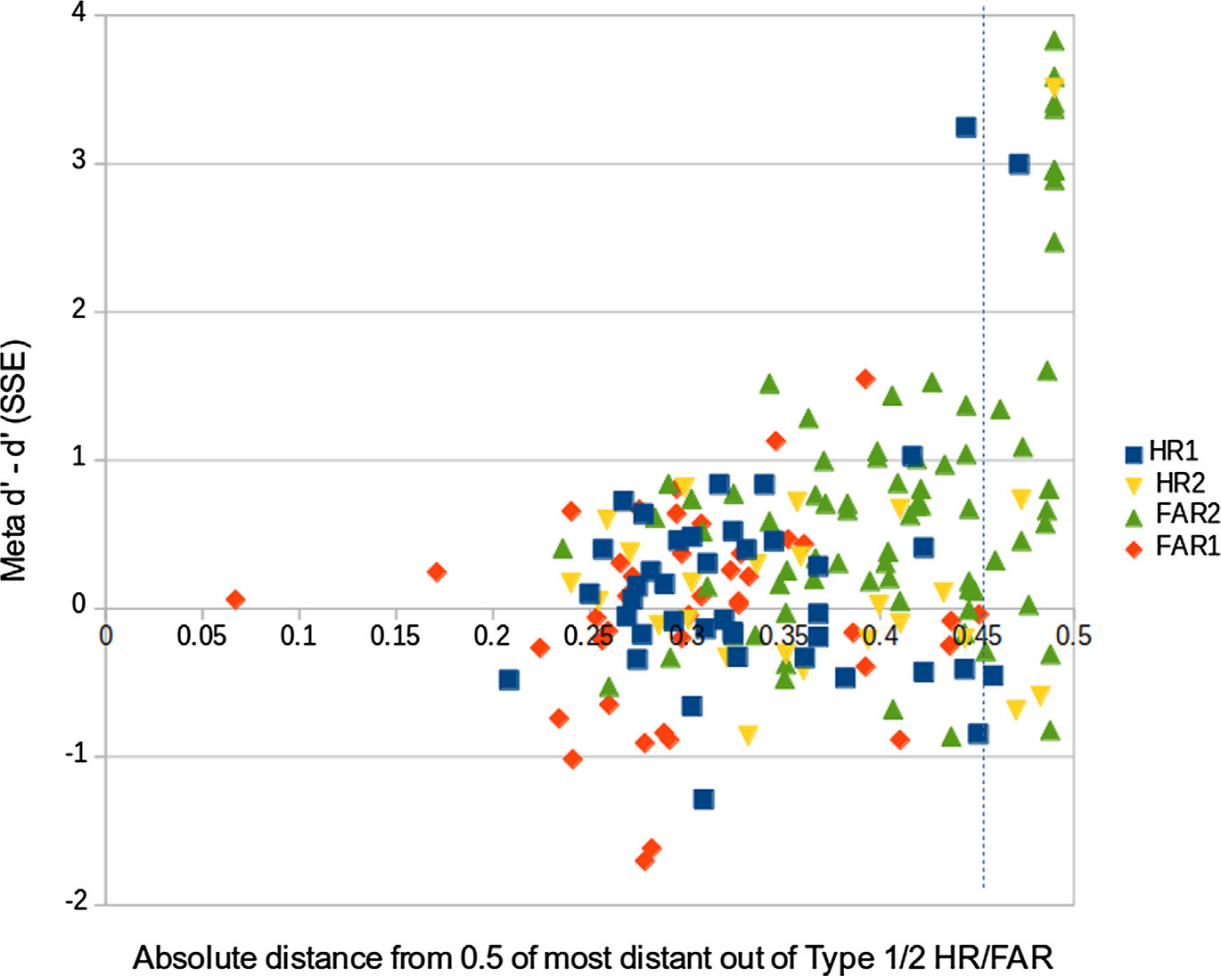
Each datapoint is the most extreme value (absolute distance from 0.5) out of 4 possible values (type I/II HR/FAR) for one session for each subject from experiment 1 of Bor et al. (2017) (180 datapoints, 90 subjects x 2 sessions). The extent of this most extreme value on the x axis (more extreme on the right) is plotted against meta d’ minus d’ (SSE) on the y axis. The vertical dotted line is the boundary by which we excluded subjects with values to the right of this in Bor et al. (2017), in order to exclude type I/II HR/FAR values too extreme to be to generate a reliable meta d’ - d’ measure.

Although the above figure was for simulated data, this is an empirical problem too. Fig. 2 below shows that, for all datapoints from experiment 1 of Bor et al. (2017) those that have more extreme (close to 0 or 1) FAR and HR values, which appear to the right of the graph, have a far larger spread of meta d’ - d’ values, as would be expected from the arguments above. In theory, meta d’ - d’ should have a maximum value of 0. In practice, this is violated when sample sizes aren’t large, and this problem becomes exaggerated when type I and II HRs and FARs are extreme. Note that the figure demonstrates that meta d’ prime values markedly above the theoretical maximum of type I d’ can occur with either extreme HR and FAR values at the type I or II level.

To put it even more bluntly, such subjects with extreme SDT values will have a meta-d’ measure that will not reflect their metacognitive performance, since the measure is effectively breaking down due to the extreme HR or FAR. These extreme meta d’ values can have an influential effect at the population level, depending on what statistical tests are used.

Ruby et al. take the view that all that matters is whether subject exclusion will affect FP (or FN) values, e.g. “we emphasize that in classical hypothesis testing there are two main types of error: Type I and Type II. Within this standard framework, any statistical decision or justification of a choice of procedure is meaningful only with respect to its impact on these two kinds of error” (p. 39). Our view instead is that the proper application of statistical techniques requires confidence that measured variables, on which inference is performed, reflect the property of interest. If it’s clear that some portion of data, due to mismeasurement (e.g. subject responses orthogonal to question of interest, or extreme HR/FAR values as in the present case) do not reflect the property of interest, then they should be excluded for this reason, regardless of statistical concerns (although usually a likely corollary is that FP or false negative (FN) rates may well be reduced). From this perspective, the main purpose of the Ruby et al. paper, namely to use simulations to demonstrate that FP rates are unaffected by subject exclusions, becomes irrelevant.

It is worth noting that these principled reasons apparently motivated the authors of the original Rounis study to mitigate these problems in their original design, by instructing subjects to keep their type II responses as even as possible, thus avoiding the extreme HR/FAR values. Having taken such clear methodological steps to reduce extreme values, it would then seem reasonable for Rounis et al. to have excluded their remaining subjects that included extreme HR/FAR values, as we did ours. However, we also note that had they done this, as Ruby et al. report, they would have reduced their n from 20 to 7. In other words, their attempt to avoid the extreme HR/FAR values by this design failed.

### 2.2 Issues with the Ruby et al. simulations

A second point of disagreement has to do with the relative value of empirical data compared to simulations. In both the Rounis et al. and Bor et al. studies, including unreliable meta d’ measures, based on extreme HR/FAR data leads to a significant result (though in both studies this was a parametric test on non-gaussian data and in our study this was the case when using only one of various metacognition measures, and only on one of two large experiments), while excluding these results leads to a null. This is entirely consistent with the notion that including unreliable meta d’ scores increases one’s chances of a false positive, which to our minds provides the most parsimonious explanation of both these results. The Ruby et al. simulations may even lend credence to this interpretation, given that they show (Table 1, supplementary materials) that power should have increased in our between subjects design (where there was a positive result on one measure without excluding subjects) after exclusion.

Principled analysis of actual empirical data is preferable to claims based on computer simulation of empirical data that are then used to reinterpret that data. Simulations necessarily have a range of parameters, some of which are inevitably set with somewhat arbitrary values, as compared to rigorous theoretical and analytical approaches applied to empirical data. In addition, simulations often embed assumptions about the nature of the data generating process which are by construction in line with the analysis methods, meaning that they will tend to overestimate the sensitivity and validity of the corresponding analyses.^2^ Accordingly, the Ruby et al. simulations make the assumption that type I and II decisions are based on the same evidence, distributed according to a standard SDT model.

Furthermore, the simulation parameters used by Ruby et al. to model our (2017) data are drawn from the original Rounis et al. study empirical data, not from the empirical data in Bor et al. As Ruby et al. themselves emphasise, the two studies are not identical (since we made some minor modifications to improve the design, as we describe in detail in our paper). In which case, it would seem more appropriate to use the parameters from our own empirical data, which are bound to differ somewhat from their dataset.^3^ The potential (and as yet unknown) impact of these choices about parameter values again underlines the potential dangers of relying on simulations as compared to principled theoretical analysis.

In addition, the analysis of Barrett et al. (2013) suggests that including extreme data points should weaken statistical inference since, as we have already noted, small random fluctuations in hit and miss rates at extreme ends of the spectrum will translate into large fluctuations in measured values of meta-d’. Ruby et al.’s simulations of Bor et al.’s Experiment 1 concord with weaker inference in this sense, but for Experiment 2 they surprisingly find the opposite, marking an intriguing discrepancy deserving of further research. One possibility to resolve this discrepancy would be to create simulations based on distributions of empirical hit and false alarm rates in Rounis et al, and use these to generate surrogate data, rather than starting from the levels of meta-d’ as currently done in Ruby et al. This approach would avoid making assumptions about how responses are generated in response to stimuli, and would require only the assumption of trial-to-trial independence.

### 2.3 Issues with the Bayesian assumptions

Ruby et al. claim that DLPFC tbTMS in the Bor et al. study actually caused a metacognitive deficit, on the basis of a Bayesian analysis, using a prior of 0.5, partially based on the Rounis et al. data. We are not compelled by this logic since we have argued that the results found in Rounis et al. are based on invalid data (i.e., data obtained without subject exclusion). It therefore seems arbitrary and unjustified to assign any Bayesian prior (including a neutral prior of 0.5) to the probability that the effect found in that study is true. Indeed, the primary contribution of our study was to examine what happens when only valid data are included in the analysis.

If a prior is anyway to be selected, what should that prior be? Based on only valid data, both the Rounis and Bor et al. studies found a null result. In addition, the Ruby et al. paper cites a relevant recent TMS prefrontal cortex metacognition paper by Rahnev and colleagues (Rahnev, Nee, Riddle, Larson, & D'Esposito, 2016), where TMS to prefrontal cortex actually *enhanced* perceptual metacognition. Ruby and colleagues conclude that these results “suggest that different parts of DLPFC perform different [metacognitive] functions.” However, given that tbTMS to both an anterior and dorsolateral region of the prefrontal cortex enhanced perceptual metacognition compared to a control site, the results of this new study are evidently relevant to any putative Bayesian prior, making a metacognitive impairment due to DLPFC TMS even less likely. If two null results and an opposite effect are input into a Bayesian prior, we assume the value would be very different to 0.5 and consequently the conclusions of the Ruby et al. paper about the likelihood of our results actually reflecting a DLPFC-induced metacognitive impairment would be very different as well.

### 2.4 Did Bor et al. unintentionally replicate Rounis et al.?

Ruby et al do not merely disagree with our conclusions in Bor et al. They in fact say, “We therefore find it striking that Bor et al.’s (2017) results appear to demonstrate a successful replication prior to subject exclusion, despite their interpreting otherwise.” Because of the contentious nature of this claim, we believe it is worth going into the details of what we did and did not find. It is true that, using one particular method of computing meta-d’ (sum-square-error, SSE), in Experiment 1, when not excluding the subjects with unreliable meta d’ scores due to extreme HR/FAR values, on a one-tailed *t*-test, without multiple comparisons on non-parametric data we found a significant difference between the DLPFC and control (vertex) group (p = 0.03). We included this invalid statistical test (a parametric test on non-parametric data) explicitly to make a point: “We should emphasise, however, that this significant result, aside from being uncorrected for multiple comparisons [we had 4 experimental groups and three metacognitive measures], is not to be trusted as it includes data that invalidates the (parametric) assumptions underlying the analysis. We merely include this analysis to demonstrate how the inclusion of unstable [unreliable] values could potentially generate spurious significant results.” (p. 12).

It is unclear whether a one-tailed *t*-test was appropriate here, given any prior expectation to replicate the Rounis et al. result was potentially nullified by other more recent studies showing the opposite results, such as Rahnev et al. (2016). Had we used a two-tailed *t*-test, this single significant result would not have been significant. Similarly, had we corrected either for the number of experimental group or number of metacognitive measures, the result would not have been significant either.

Our suspicion was that the main parametric result from Rounis et al. was based on non-normal data, as was our significant result above. Had we carried out the more appropriate non-parametric equivalent test, this result would not have approached significance (Wilcoxon Rank Sum Test W = 113, p = 0.1949). Ruby et al. confirm the non-normality of their original data in the last paragraph of their supplementary materials document, and claim that the critical non-parametric equivalent interaction test was still significant, though unfortunately without providing any details on p-values or related quantities.

Moreover, in the Bor et al. case the effect (if any) seems to be driven by an unpredicted boost to metacognition in the control group, rather than a reduction in metacognitive performance in the experimental (DLPFC) group, as interpreted by Rounis et al. This solitary statistical effect therefore does not seem to replicate Rounis et al., contrary to Ruby et al.’s claim.

In addition, when we used the main alternative version kindly supplied by Rounis and colleagues (maximum likelihood estimation, MLE), or a new Bayesian method by Stephen Fleming^4^ (HMeta-d), without exclusion, there was also no such significant effect (with unreliable measures: MLE p = 0.66, HMeta-d p = 0.65; excluding unreliable measures MLE p = 0.69, HMeta-d p = 0.54). We therefore disagree that our study constitutes an unintended replication of Rounis et al., especially when taken in combination with the rest of our analyses which rigorously failed to find evidence for any effect, including Bayes Factor robust findings for a null.

Furthermore, in Experiment 2 within the Bor et al. paper, we were even less successful in attempting to replicate the Rounis et al. findings. We designed our Experiment 2 as an adaptation of a within-subject design, and was therefore in this way a closer replication of Rounis et al. Our double-within-subjects protocol (potentially having 4 sessions of TMS and testing, instead of 2) was in fact designed to be sensitive to the presence of even just a single subject (out of the original 27) who would follow the pattern of the Rounis et al. study. Had we found just one subject who replicated the Rounis et al. results over the 4 sessions of session 2, we may have been able to describe this experiment as providing a partial replication. This was indeed our intention, contrary to Ruby et al. in their abstract claiming that we “adopted an experimental design that reduced their chance of obtaining positive findings.”

However, we did not find even a single subject who followed such a pattern. The first session involved tbTMS to DLPFC for all 27 subjects. Of those 27, 10 were excluded due to extreme SDT values. Out of these 17 subjects, 7 showed an effect (in either direction) equivalent to the Rounis et al. study. Our original wording from Bor et al. makes clear the nature of our non-replication: “Of the remaining 7 participants, 3 showed the expected impairment, while 4 showed a clear metacognitive enhancement following DLPFC cTBS. 6 of these 7 participants also showed a clear metacognitive change for the vertex control session, and thus were not asked to return for the 3rd session (2nd DLPFC). Only 1 participant that showed a clear DLPFC cTBS metacognitive change in the first session also showed no change for the 2nd vertex cTBS session, and thus was brought back for the 3rd session (2nd DLPFC). This session, unfortunately, included unstable SDT values [here meaning extreme HR/FAR values close to 0 or 1], and thus the participant was not asked to return for a 4th session. If these instabilities [unreliable measures] are ignored, though, the metacognitive change for the 3rd session was very similar to the 1st session. Both sessions, however, showed a robust *enhancement* of metacognition for this single subject following DLPFC cTBS, as opposed to the impairment found in the Rounis study.” (Bor et al., 2017, p. 13).

A comprehensive appreciation of our Experiment 2 therefore reveals that not even a single participant replicated the results in Rounis et al. Even when just considering the first two sessions, which would make our Experiment 2 even more closely resemble the original Rounis et al. study with its two sessions, not a single participant (out of 27) showed both a DLPFC tbTMS metacognitive impairment, as well as *no* metacognitive effect when tbTMS is applied to the control site (vertex).

In terms of statistical power, which Ruby et al. critique for our Experiment 2, we note that their reported power for our Experiment 2 after excluding subjects was 0.308, which was almost identical to the Rounis et al. study (without subject exclusions) of 0.311. (The greatest statistical power across both the Rounis et al. and Bor et al. experiments was in our Experiment 1 after excluding subjects: 0.409.) Thus, on the basis of power calculations, it remains difficult to explain why our experiments failed to find a positive result, while the Rounis et al. study did find such a result. However, we emphasise that modelling of power and false positive rates are a poor alternative to rigorous analysis of the empirical data itself, as our Experiment 2, in particular, demonstrates.

Finally, even if discounting all the arguments we make above, relying on outliers for a positive result – as in the original Rounis et al. study – suggests, at best, only a small effect size.

### 2.5 Design differences between the Bor et al. and Rounis et al. studies

In their manuscript, Ruby et al. also criticise the fact that we modified the experimental design of Rounis et al. in various ways. For example, they say “[Bor et al.] made several changes to the original study design, some of which are known to undermine the chance of finding meaningful results from the outset”.

Indeed, we did make small changes to the Rounis et al. design. As we made clear in Bor et al., in each case the changes were made to improve the design with respect to its suitability to address the underlying experimental question. Some of these changes should be entirely uncontroversial; for instance, in removing some minor software ‘bugs’ in the experimental scripts which Rounis and colleagues kindly shared with us, and in ensuring consistency in behavioural responses, and so on (see Bor et al., p. 3).

One notable change was that we used a between-subjects design rather than (as in Rounis et al.) a within-subjects design for our Experiment 1. This was mandated by our original aim to replicate and extend Rounis et al. by exploring the potential causal contribution of other regions within the frontoparietal network to metacognition. Our Experiment 2, however, utilised a double-repeat within-subject design and still failed to find any effect of cTBS on metacognition. This double-repeat design exemplifies that the changes we made were employed to maximize the chance of finding a true effect should one exist.

The other design changes in Bor et al. (2017) were also made in a principled way, and captured in full the spirit of the original Rounis et al. experiment. These changes are fully described in Bor et al. (see, for example, p. 15) and we will just reinforce two points here. First, there were small changes to task instructions: we used confidence rather than visibility ratings (to ascertain metacognition more directly and to avoid additional working memory demands). However, in attempting to avoid the issues around relative, rather than absolute, metacognitive judgements, of the original Rounis study, we recognise that asking a different metacognitive question could have led to different results. Whether such a subtle change is sufficient to turn a significantly positive finding into a null result is a question we address in the next section.

We also used an active TMS control (vertex) rather than sham TMS, to provide a closer match between experimental and control conditions. Part of our justification for this change was to address demand characteristics, with sham TMS versus real TMS potentially causing subjects to demonstrate a stronger impairment for that condition (DLPFC) for which they perceive is the real condition. However, although we continue to believe that a real TMS site is preferable to sham as a control, we also acknowledge that the choice of vertex itself is not perfect. First, a vertex control would not have induced as many peripheral nerve issues (facial twitching and pain) as the DLPFC stimulation in a subset of subjects (though would be similar to the parietal TMS stimulation we used in experiment 1). Second, the vertex control meant that the same region was stimulated twice, unlike any of the experimental conditions. It is difficult to know how such a difference would affect participants both psychologically and neurophysiologically.

Interestingly, at one point, Ruby et al. (p. 15) criticise that we used only two levels of confidence judgements, suggesting that using more than two would have allowed more sensitive estimation of meta-d’. However, although we agree that more than two levels would have been beneficial,

we used two levels because Rounis et al. also used two levels (in their case of visibility ratings rather than confidence). Had we used more than two levels, this would have changed the design considerably, and any subsequent null result might have been attributed to such a clear departure from the original protocol.

### 2.6 What constitutes a good replication

These points speak to a broader discussion of what counts as an adequate replication in cognitive neuroscience [see, for example, (Szucs and Loannidis, 2017)]. Our view is the following: Replications in cognitive neuroscience are not (yet) like replications in experimental physics – cognitive neuroscience (broadly construed) necessarily embeds a greater degree of flexibility in how experiments are designed and conducted, given the range of theoretical views, experimental methods, and analysis techniques. In addition, for any particular experimental result to be described as ‘strong’, it should be robust to small variations in paradigm, especially when such variations should be expected to improve the design according to principled criteria.

A related point is that, as Ruby and colleagues admit, both the original Rounis et al. and the Bor et al. studies were relatively underpowered. We attempted to include a high n and sufficient power in our studies, but the extent of subject exclusions due to extreme HR/FAR values was substantial, which weakened power, even when the data were cleaner. Our view is that the best way forward is to collect more empirical data, rather than run simulations. We freely admit limitations in both the Rounis and Bor et al. studies, and that a new experiment with more robust power could address these limitations. A new design could also improve on both studies by having MRI-guided TMS, using a continuous confidence or visibility judgement (or at least including many more options than the binary options we both used), with a sufficiently large n, and multiple controls (e.g. both sham and real TMS at various non-DLPFC sites). To our minds, such a study could definitively answer the question of whether tbTMS to DLPFC can impair visual metacognition, and would be far beneficial to any simulation or to debates about the perceived differences between two experiments.

In general, lack of power is a persistent problem in cognitive neuroscience studies (Szucs and Loannidis, 2017), and if this is combined with the bias towards positively significant results, creates an atmosphere where Szucs and Loannidis (2017) have concluded that “more than 50% of published findings deemed to be statistically significant are likely to be false.” Consequently, both the original study, and the attempted replication, should strive for high statistical power. Furthermore, given the issue of failed replications, we believe that there should be a greater onus on initial publications to demonstrate robust effects, both in terms of power and the extent of the robust statistical findings.

### 2.7 Is the prefrontal cortex involved in metacognition?

Ruby et al. imply that we argue against any role for PFC in metacognition or conscious perception. For instance, in the introduction they write that following our null result, “they… suggest that DLPFC might not be ‘critical for generating conscious contents’.” This quoted phrase is a misrepresentation of our position, and we urge readers to read the entire discussion from Bor et al. to retrieve the full context. There we raised the possibility that our findings in Bor et al. are consistent with so-called ‘no report’ paradigms which have independently cast doubt on the role of the DLPFC in generating conscious contents (e.g. Brascamp, Blake, & Knapen, 2015), but also raised other possibilities, such as that the prefrontal parietal network might be especially plastic, and adapt within seconds to TMS administration, or that tbTMS to DLPFC at ethically safe levels simply isn’t sufficient to induce an impairment in a subtle paradigm. We nowhere deny that there is other evidence, from other studies (including, for example, lesion studies) that speak to a role for PFC in metacognition, or indeed in generating conscious contents more generally (Bor, 2016).

Indeed, we completely agree with Ruby et al. when the say “Ultimately, whether theta-burst TMS to DLPFC can robustly impair visual awareness concerns the specific method and details. More important is the general question regarding the role of the prefrontal cortex in metacognition and conscious perception”. Absolutely. But the only points of debate between Ruby et al. and Bor et al. concern the specific details and methods. Simply citing evidence from other studies relating to PFC and consciousness does not contribute to the present discussion; instead it falsely suggests that one should believe Rounis et al. simply because it may possibly fit better with some other independent experiments. However, Ruby et al. agree with us that establishing the role of PFC in awareness is a very important, interesting, and open question (Bor and Seth, 2012).

## 3 Conclusions

In summary, we continue to think the evidence so far, across studies, does not (so far) demonstrate that theta-burst TMS to prefrontal cortex can disrupt visual metacognition. Moreover, the findings from each study depend on the fine details of methods and analyses. Further investigations would be helped by open access provision of data (as done for Bor et al., but not for Rounis et al.), and simulation code (used in Ruby et al.) – so that interested researchers can draw their own conclusions in addition to the opinions presented in this series of papers. We also look forward to further studies with increasingly refined methodologies, motivated by the discussions here.

## Footnotes

1 We also excluded smaller numbers of subjects for other reasons: equipment failure (n = 7), insufficient behavioural accuracy (n = 3) (for our Experiment 1).

2 As an example, consider simulations of interacting variables used to test properties of Granger causality. Most such models (e.g., Seth, 2010) make use of the same autoregressive models that are employed in Granger causality analysis itself, biasing these simulations towards favouring the analysis. Only a minority deliberately use generative simulation models which are explicitly distinct from the statistical model assumed by the analysis method [see, for example, (Seth, Chorley, & Barnett, 2013)]. A similar story can be told for the effective connectivity method of ‘dynamic causal modelling’ (Friston, Moran, & Seth, 2013).

3 For instance, the Ruby et al pre-TMS d' mean parameter was 1.67, compared to 1.37 in our case – a considerable difference.

4 https://github.com/metacoglab/HMeta-d.
